# Effectiveness of a Mobile Device–Based Resilience Training Program in Reducing Depressive Symptoms and Enhancing Resilience and Quality of Life in Parents of Children With Cancer: Randomized Controlled Trial

**DOI:** 10.2196/27639

**Published:** 2021-11-29

**Authors:** Yuanhui Luo, Wei Xia, Ankie Tan Cheung, Laurie Long Kwan Ho, Jingping Zhang, Jianhui Xie, Pin Xiao, Ho Cheung William Li

**Affiliations:** 1 School of Nursing The University of Hong Kong Hong Kong China; 2 School of Nursing Sun Yat-Sen University Guangzhou China; 3 Nethersole School of Nursing The Chinese University of Hong Kong Shatin, New Territories Hong Kong China; 4 Xiangya School of Nursing Central South University Changsha China; 5 Department of Nursing Hunan Children’s Hospital Changsha China; 6 Department of Hematology Hunan Children’s Hospital Changsha China

**Keywords:** depressive symptoms, pediatric cancer, parents, quality of life, resilience, mobile phone

## Abstract

**Background:**

Caring for children with cancer can be a stressful experience for parents and may have negative effects on their physical and psychological well-being. Although evidence has shown that resilience is associated with positive psychological well-being, few interventions have been specifically designed to enhance the resilience of parents of children with cancer.

**Objective:**

The aim of this study is to examine the effectiveness of a mobile device–based resilience training program in reducing depressive symptoms and enhancing resilience and quality of life (QoL) in parents of children with cancer.

**Methods:**

Parents of children diagnosed with cancer were recruited from the pediatric oncology wards of 3 tertiary hospitals in China. The participants were randomly assigned to either the experimental group (52/103, 50.5%) to undergo an 8-week mobile device–based resilience training program or to the control group (51/103, 49.5%) to receive an 8-week program of placebo information. The study outcomes included resilience, depressive symptoms, and QoL, as measured by the Connor–Davidson Resilience Scale, the Self-Rating Depression Scale, and the Short Form of the 6-Dimension Health Survey, respectively. All data were collected at baseline and at 2 and 6 months of follow-up. The data analysis followed the intention-to-treat principle. A generalized estimating equation was used to examine the effects of the intervention.

**Results:**

The participants were mostly female (72/103, 69.9%), and their mean age was 33.6 (SD 5.2) years. The participants in the experimental group showed significantly higher levels of resilience (mean 67.96, SD 15.8 vs mean 58.27, SD 19.0; *P*<.001) and lower levels of depressive symptoms (mean 40.17, SD 9.9 vs mean 46.04, SD 10.9; *P*<.001) than those in the control group at 6 months of follow-up. The intervention showed statistically significant effects in improving resilience (*β*=6.082; *P*=.01) and decreasing depressive symptoms (*β*=−2.772; *P*=.04) relative to the control group. The QoL score in the experimental group was higher than that in the control group at 6 months of follow-up (mean 0.79, SD 0.2 vs mean 0.76, SD 0.3; *P*=.07); however, no statistically significant intervention effect was detected (*β*=.020; *P*=.38).

**Conclusions:**

The mobile device–based resilience training program effectively enhanced resilience and alleviated depressive symptoms in parents of children with cancer. It is highly recommended that health care professionals incorporate this resilience training program when providing psychological care to parents of children with cancer.

**Trial Registration:**

Clinical.Trials.gov NCT04038242; http://clinicaltrials.gov/ct2/show/NCT04038242

## Introduction

### Background

Approximately 300,000 new cases of childhood cancer are diagnosed annually worldwide [1]. The incidence of childhood cancer in China is similar to that of the world and has increased at a rate of 2.8% per year [2]. The diagnosis of cancer in children and long-term treatment are not only stressful events for the child but also disrupt the parents’ daily lives, especially within the first year after the diagnosis, which has been described as the most chaotic moment of the parents’ lives [3]. Parents must reorganize their family roles and routines, learn cancer-related information and care skills, prepare for overwhelming medical expenses, and manage intensive treatment regimes, each of which can cause tremendous stress and affect the parents’ physical and psychological well-being [4].

Of all the negative psychological consequences, depressive feelings are one of the most common concerns reported by parents of children with cancer [5,6]. A recent meta-analysis found that the prevalence of depressive symptoms among parents of children with cancer varied from 7% to 91%, with a pooled prevalence of 28% [5]. A cross-sectional study of Chinese parents of children with leukemia found that as many as 77% of mothers and 42% of fathers had depressive symptoms [7]. Although the negative effects of parents’ depressive symptoms on the quality of life (QoL) and well-being of both the parents and their children with cancer are widely acknowledged [8,9], little attention has been devoted to the prevention and alleviation of depressive symptoms in Chinese parents of children with cancer.

Resilience is the process of adapting well in the face of stress or adversity; it involves personal virtues and strengths that can be accessed and cultivated to achieve growth under detrimental conditions [10]. Evidence has shown that resilience is associated with psychological well-being in both clinical and nonclinical populations [11,12]. A strong negative relationship between resilience and depressive symptoms has also been found in parents of children with cancer [13]. It has been suggested that resilience can play an important role in protecting individuals from stress-related disorders [14]. Therefore, interventions that enhance resilience in the parents of children with cancer may influence their depressive symptoms and well-being.

A recent meta-analysis revealed that resilience training programs that aim to equip individuals with the resources and skills to navigate adversity and thrive in challenging environments had a moderately positive effect on the subjects’ resilience [15]. These resilience training programs tend to include a combination of evidence-based training in areas such as cognitive strategies and mindfulness [15]. A review of the literature shows that most resilience training programs target students and patients with chronic illnesses [16]. No study has yet examined the effectiveness of resilience training programs in promoting the psychological well-being of parents of children with cancer. A study in the United States demonstrated the moderate effectiveness of a resilience training program delivered face-to-face to the parents of children with serious illnesses [17]. However, face-to-face training is time-consuming and requires frequent visits, which often leads to low compliance [15]. It has also been reported that some parents of children with cancer have less motivation to attend face-to-face training because of their busy schedules [18]. Mobile health (mHealth), which refers to health practices conducted via mobile devices, is increasingly common and has gained support from the World Health Organization for health promotion and treatment compliance [19,20]. It has also been suggested that internet- and mobile-based psychological interventions are cost-effective for both psychiatric and medical conditions [21]. The use of mobile apps to deliver resilience training has certain advantages; for example, health care professionals can give the participants remote support and feedback to promote their psychological well-being. Most importantly, training via mobile apps is more flexible and efficient than face-to-face interventions [22], particularly during a pandemic, when the delivery of face-to-face health care interventions may not be feasible. It is worth noting that with the rapid development and high use rate of WeChat (Tencent Holdings Limited) [23], such mobile apps may have the potential to deliver health interventions, increase adherence to training, and serve a large number of participants. This study aims to explore the effectiveness of a mobile device–based resilience training program in reducing depressive symptoms and enhancing the resilience and QoL of parents of children with cancer.

### Theoretical Framework

The training program used in this study was guided by the resilience framework developed by Kumpfer [24]. The resilience framework generated 5 internal resilience factors: cognitive, emotional, spiritual, behavioral, and physical. Specifically, cognitive competency can help individuals think rationally and protect them from negative thoughts; emotional stability can help individuals recognize and deal reasonably with negative emotions; an individual’s spiritual belief in his or her ability to improve the situation and achieve goals can help them survive difficult times; behavioral skills such as problem-solving can increase an individual’s self-efficacy to tackle life’s problems; and individuals with few physical problems may internalize their physical strength and interpret themselves as being psychologically healthy [24]. These internal resilience factors represent fundamental elements that have been shown to be essential to cope effectively with life stressors and to adapt well in the face of adversity [10]. Therefore, by cultivating the skills and resources to foster these internal resilience factors, we expect that the intervention would promote parental resilience and improve parents’ well-being.

## Methods

### Trial Design

This study was a 2-arm, parallel-group, randomized controlled trial (RCT) that adheres to the CONSORT (Consolidated Standards of Reporting Trials) guidelines [25]. This study was approved by the institutional review board of the University of Hong Kong/Hospital Authority Hong Kong West Cluster (UW 19-436) and registered at ClinicalTrials.gov (NCT04038242).

### Participants

From August 2019 to July 2020, the parents of children who received a diagnosis of cancer in the pediatric oncology wards of 3 tertiary hospitals in China and who met the following inclusion criteria were invited to participate in this study: (1) a child (aged 0-19 years) in whom cancer was diagnosed within the past year, (2) the ability to read Chinese and speak Mandarin, and (3) a smartphone with the WeChat app. Parents with cognitive impairments or physical disabilities identified from medical records and those who were participating in other psychological interventions or consultations were excluded.

Power analysis using G*Power 3.1 was performed to estimate the sample size [26]. According to a previous study that explored the effectiveness of a resilience training program in parents of children with serious illnesses [17], the effect size for resilience was 0.59. Thus, to predict the difference between the 2 groups at a 5% level of significance and a power of 0.8, 37 subjects were required for each group. Given a potential attrition rate of 20%, an additional 10 subjects were needed for each group. Therefore, the total sample size for this study was 94, with 47 subjects in each group.

### Randomization and Blinding

Simple randomization was used in this study. The participants were randomly allocated to the experimental group or control group in a 1:1 ratio. Random numbers were generated using IBM SPSS Statistics (version 25.0, IBM Corp) before recruitment. To guarantee allocation concealment, an independent researcher who was not involved in the recruitment matched the random numbers with the participants in chronological order of recruitment based on a WeChat official account follow list. Single blinding was used, and the research assistants who collected the data were blinded to the participants’ group allocation.

### Intervention

All participants were required to scan a quick response code to follow the WeChat official account at enrollment to participate in the intervention. The WeChat official account is the equivalent of a Facebook page and synthesizes functions, including tweet design and instant interaction communication [27]. The public can receive tweets after following the official WeChat account.

**Figure 1:**
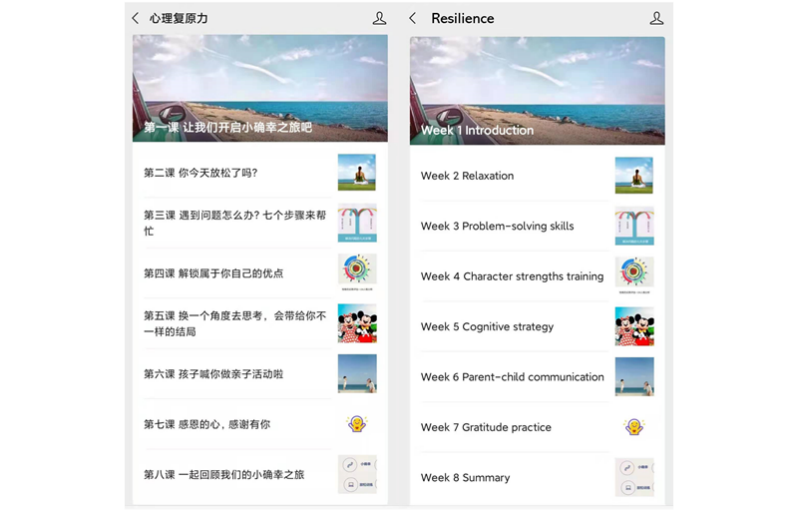
The total 8 tweets in the mobile device–based resilience training program.

The participants in the experimental group participated in the resilience training program with 8 tweets that focused on the cultivation of internal resilience factors that enhance parental resilience (Figure 1). Each tweet session was constructed in 3 parts, including detailed skill training methods with pictures or short videos, examples of applying the skills when caring for children with cancer, and an assignment to reinforce the training (Multimedia Appendix 1). The participants were required to read the tweet sessions and finish the web-based assignments, which took approximately 15 minutes for each training session. Feedback on the assignment from a psychological consultant was sent back to the participants via WeChat within 1 working day. We sent the tweets weekly on each Saturday at 8 PM, and a reminder of the assignment was sent at the same time the next Wednesday to promote the participants’ enrollment in training. To ensure that the intervention had an adequate effect on the outcome measures of this study, the dosage was assessed in terms of amount, frequency, and duration by a research committee that comprised a professor of psychology and an associate professor of psycho-oncology with extensive experience in psychological interventions, a professor of pediatric oncology and 2 senior pediatric oncology nurses with considerable clinical experience, and a research fellow with rich experience conducting resilience training programs. The feasibility and acceptability of the interventional dosage and content were evaluated in a 1-group pilot trial with 10 participants. Most of the participants commented that the length and frequency of the tweets were acceptable, and they were able to comprehend the content of the tweets. Hence, no changes were made to the content of the intervention. To control for potential contamination in the study, we set up the tweets to be sent only to specified participants, and the tweets could not be shared. Table 1 presents the detailed content of the mobile device–based resilience training program.

The participants in the control group received 8 tweets that contained information from a caring manual distributed by the hospital to every child with cancer. The content of the tweets included oral care, symptom management, peripherally inserted central catheter maintenance, dietary guidance, medication care, knowledge about bone marrow biopsy, and infection prevention. The number and frequency of tweets were the same as those in the experimental group. No assignments or feedback were delivered to the participants in the control group.

Several strategies were adopted to ensure the integrity of the intervention. First, a research protocol was developed, and the interventions strictly followed the protocol. Second, the number of tweets and feedback sent to each participant was recorded in Microsoft Excel (Microsoft Corporation) to monitor the delivery of the interventions. In addition, team meetings were held monthly to evaluate the quality of the intervention’s implementation.

**Table 1:** Content of the mobile device–based resilience training program.

Sessions or tweets and internal resilience factors			Details				
			Objectives		Core content		Assignment
**Week 1: introduction**							
	Emotional	To help the participants understand the purpose of the intervention To help the participants initially understand the intervention content		Definition of resilience, purpose of the study, science of the intervention, general content of all tweets, and encouragement to cultivate positive emotions in daily life		The 3 good things practice: write down 3 things that go well for you that day and reflect on why they went well	
**Week 2: relaxation**							
	Emotional and physical	To practice relaxation techniques To reduce the participants’ stress		Definition of meditation, science and specific practice methods of meditation exercise, and guided meditation audio		Guided breath awareness: sit quietly and be aware of your breath and exercise following the guided meditation audio	
**Week 3: problem-solving skills**							
	Behavioral	To learn problem-solving skills To cultivate a positive coping style		Introduction and science of the problem-solving therapy, specific 7 steps to solve problems, and examples of solving problems encountered in caring for children with cancer		Problem-solving practice: complete the problem-solving worksheet according to the instructions	
**Week 4: character strength training**							
	Cognitive	To help the participants understand their own character strengths To increase confidence in dealing with difficulties in life		Definition of character strength, establishment of character strength assessment system, and science and specific methods of character strength training		Character strength training: finish web-based character strength assessment to find your top 5 strengths. Use your strengths in caring for your child	
**Week 5: cognitive strategies**							
	Cognitive and emotional	To help the participants reframe negative or unhelpful cognition To learn emotion management skills		Recognize and accept emotions, definition of cognition, science of cognition restructuring, examples of cognition restructuring scenarios from parents of children with cancer, and emotion management strategies		Cognition restructuring practice: complete the cognitive reconstructing worksheet according to the instructions	
**Week 6: parent–child communication**							
	Behavioral	To promote effective communication between parent and child To help the participants build a good relationship with their child		Ways to achieve effective parent–child communication, examples of good communication from parents of children with cancer, and tips for managing children’s emotions		Parent–child activity: accompany the child for an appropriate activity and apply the communication skills in the activity	
**Week 7: gratitude practice**							
	Spiritual	To help the participants cultivate positive beliefs To help the participants attain personal growth		Manifestations, benefits, science and specific exercise of gratitude; steps to make a realizable goal; and examples of goal setting from parents of children with cancer		Gratitude activity: write a gratitude letter or keep a gratitude diary or prepare a gratitude card	
**Week 8: summary**							
	Cognitive	To review the learned skills and related assignments To help the participants make a plan to reinforce the learned skills in their future life		Simple summary for each tweet, emphasis of the learned resilience skills and related assignments, and encourage the participants to make a plan to practice the learned skills		Plan-making: make a plan to practice all skills and choose a favorite exercise to keep up	

### Outcomes and Measures

At baseline, the participants’ demographic information and their children’s clinical characteristics were collected using a demographic information sheet. The questionnaire requested information about the participants’ gender, age, marital status, educational attainment, and monthly income. The participants also reported their child’s age, type of cancer, time since diagnosis, and risk stratification of cancer. In resilience intervention studies, it has been suggested that a posttest at the end of the training program and a follow-up assessment at 6 months are necessary to evaluate the efficacy of the interventions [28]. Hence, the outcomes in this study were assessed at baseline and at 2 and 6 months after the intervention began.

The primary outcome was resilience, which was measured at 6 months using the Connor–Davidson Resilience Scale, a self-rated scale designed by Connor and Davidson [29]. The Chinese version was translated and tested by Yu and Zhang [30] and has shown good reliability and satisfactory validity. It consists of 25 items and is scored on a 5-point Likert scale (0-4). The total score ranges from 0 to 100, with a higher score reflecting a higher level of resilience. It has been widely used in clinical practice and treatment-outcome research [31].

The secondary outcomes were depressive symptoms and QoL at 6 months of follow-up and resilience, depressive symptoms, and QoL at the 2-month follow-up visit. The Self-Rating Depression Scale was used to measure participants’ depressive symptoms during the previous week [32]. It includes 20 items, and half of the items are scored in reverse. The frequency of the symptom in each item was evaluated on a 4-point Likert scale (1=never or seldom, 2=sometimes, 3=often, and 4=always). The total score ranges from 20 to 80, and a higher total score indicates worse depressive symptoms. The Chinese version of this scale has shown good reliability and satisfactory internal consistency [33].

The Short Form of the 6-Dimension Health Survey was used to measure the participants’ QoL. It was developed by Brazier et al [34] and is derived from the 36-item Short Form Health Survey. Hong Kong scholars translated it into Chinese and produced a scoring algorithm [35]. This scale is a 6-dimension health survey that assesses physical functioning, role limitations, social functioning, pain, mental health, and vitality. The total score based on the scoring algorithm ranges from 0.32 for worst health to 1 for full health. The scale has been shown to be reliable and valid in a Chinese population [36].

### Data Collection

To identify potential participants, posters with information about the study were posted on the notice board in the hospitals’ pediatric oncology wards and outpatient clinics. Parents who were willing to participate scanned a quick response code on the poster to complete a simple application form. The research assistant then contacted the person who filled out the application form after assessing their eligibility. The research assistant also visited each ward and invited parents of children with cancer to participate. The study details, including purpose, procedures, and potential benefits and harm, were explained, and the parents were given the option of participation or refusal. The parents were also told that their participation was voluntary and that they had the right to withdraw at any time without reprisal. Written informed consent was then obtained from parents who agreed to participate. The baseline and 2-month follow-up data were collected using written questionnaires in the wards of the hospitals. Outcome measures at the 6-month follow-up were collected using web-based questionnaires.

### Statistical Analysis

IBM SPSS Statistics 25.0 (IBM Corp) was used for data analysis. The intention-to-treat principle was applied, and the participants were analyzed according to their initial group assignments. Missing data were handled with multiple imputation using the Markov chain Monte Carlo method. Complete case analysis was conducted to test the robustness of multiple imputation. Descriptive statistics were applied to calculate the mean and SD for continuous data and the frequency and percentage for categorical data. The range and median values for the study outcomes were displayed as a complement. The differences in the baseline characteristics and outcomes between the experimental and control groups were examined using a chi-square test or Fisher exact test for categorical variables and 2-tailed independent-sample *t* test and Wilcoxon signed-rank test for continuous variables. A generalized estimating equation was used to estimate the effects of the intervention on all outcomes with repeated measures [37]. Statistical significance was set at *P*<.05.

## Results

### Participants Characteristics

**Figure 2:**
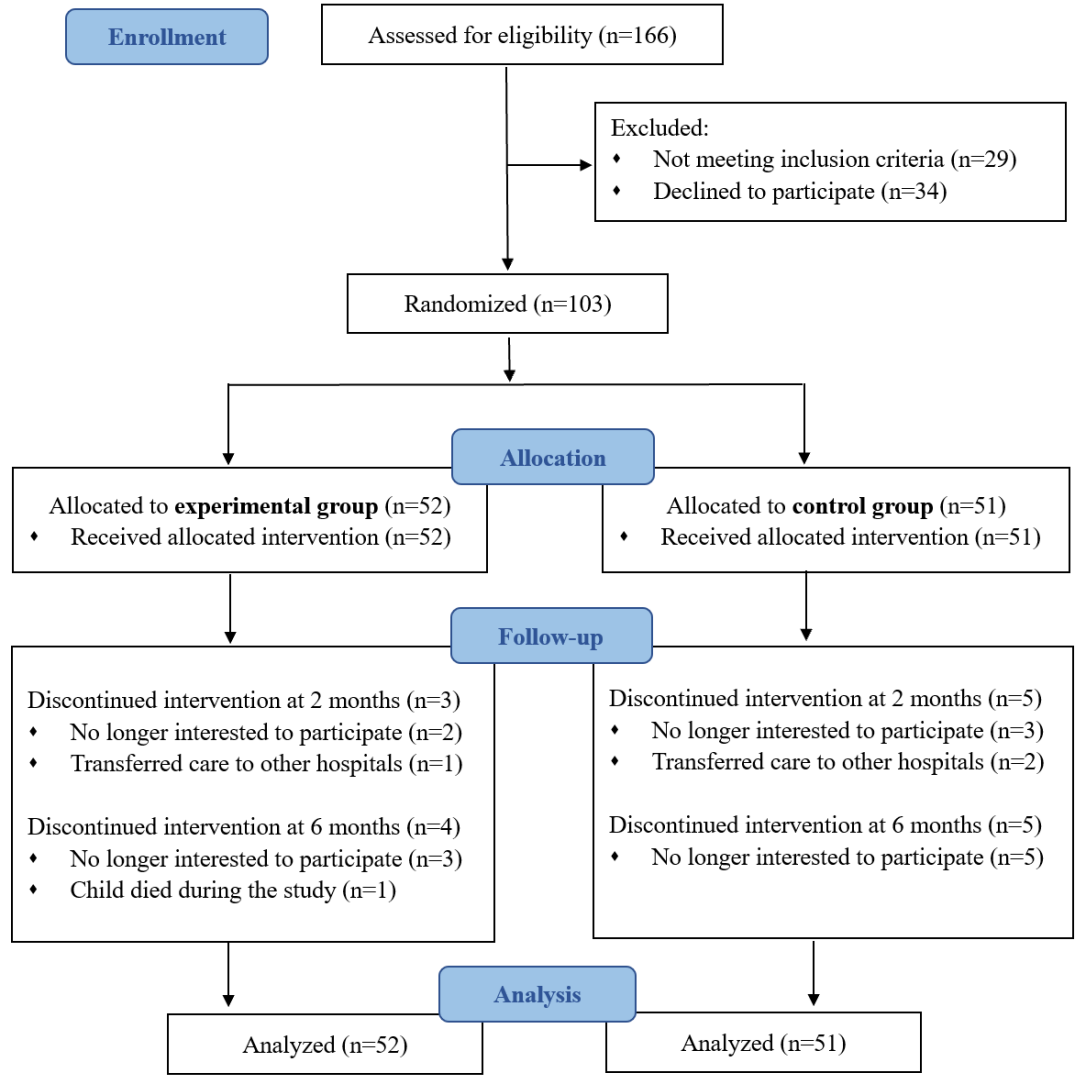
A CONSORT (Consolidated Standards of Reporting Trial) flow diagram.

A total of 103 participants were recruited; of the 103 participants, the experimental and control groups had 52 (50.5%) and 51 (49.5%) participants, respectively. Figure 2 presents the flowchart of the study. The overall attrition rate was 16.5%, and no significant differences were found in the baseline characteristics between the participants who withdrew and those who remained.

The participants were mostly female (72/103, 69.9%) and married (100/103, 97.1%), and their mean age was 33.6 (SD 5.2) years. Approximately one-third (29/103, 28.2%) had attained tertiary education, and half (52/103, 50.5%) had a monthly income of <Chinese ¥3000 (US $427.35). Approximately two-thirds (69/103, 67%) of the participants’ children received a diagnosis of a hematologic tumor, and the remaining one-third (34/103, 33%) had a solid tumor. The children’s mean age was 5.9 (SD 3.9) years, and most had received their diagnosis <6 months earlier (85/103, 82.5%). No significant differences were detected in the baseline characteristics between the experimental and control groups (*P*>.05); details are given in Table 2.

**Table 2:** Baseline characteristics of the participants in the experimental and control groups (N=103).

Characteristics			Experimental (n=52)				Control (n=51)				Chi-square (*df*)		*t* test (*df*)^a^		*P* value
			Participant, n (%)		Values, mean (SD)		Participant, n (%)		Values, mean (SD)						
**Gender**					N/A^b^				N/A				N/A		
	Female	35 (67)				37 (73)				0.3 (1)				.56	
	Male	17 (33)				14 (23)				0.3 (1)				.56	
**Marital status**					N/A				N/A				N/A		
	Married	50 (96)				50 (98)				0.3 (1)				.99^c^	
	Divorced	2 (4)				1 (2)				0.3 (1)				.99^c^	
**Educational attainment**					N/A				N/A				N/A		
	Primary school	5 (10)				3 (6)				1.1 (2)				.60^c^	
	High school	31 (60)				35 (69)				1.1 (2)				.60^c^	
	College	16 (31)				13 (25)				1.1 (2)				.60^c^	
**Monthly income (Chinese ¥; US$)**					N/A				N/A				N/A		
	<3000 (<427.35)	24 (46)				28 (55)				1.2 (2)				.54	
	3000-5000 (427.35-712.25)	17 (33)				16 (31)				1.2 (2)				.54	
	>5000 (>712.25)	11 (21)				7 (14)				1.2 (2)				.54	
**Type of cancer**					N/A				N/A				N/A		
	Hematology tumor	38 (73)				31 (61)				1.8 (1)				.19	
	Solid tumor	14 (27)				20 (39)				1.8 (1)				.19	
**Time since diagnosis (months)**					N/A				N/A				N/A		
	0-6	44 (85)				41 (80)				0.3 (1)				.57	
	7-12	8 (15)				10 (20)				0.3 (1)				.57	
**Risk stratification of cancer**					N/A				N/A				N/A		
	Low	18 (35)				17 (33)				1.0 (2)				.61	
	Intermediate	22 (42)				18 (35)				1.0 (2)				.61	
	High	12 (23)				16 (31)				1.0 (2)				.61	
Parents’ age in years			N/A		33.92 (5.4)		N/A		33.22 (5.0)		N/A		−0.691 (101)		.49
Children’s age in years			N/A		5.48 (3.7)		N/A		6.41 (3.6)		N/A		1.278 (101)		.20

^a^The significance of *t* test is 2 tailed.

^b^N/A: not applicable.

^c^Fisher exact test.

### Comparisons Between Experimental and Control Groups

Table 3 presents comparisons of resilience, depressive symptoms, and QoL between the 2 groups at each measuring point. Compared with the participants in the control group, those in the experimental group showed statistically higher levels of resilience at 2 months (mean 61.90, SD 14.6 vs mean 69.35, SD 13.4; *P*=.005) and 6 months (mean 58.27, SD 19.0 vs mean 67.96, SD 15.8; *P*<.001) and lower levels of depressive symptoms at 2 months (mean 44.66, SD 8.0 vs mean 40.40, SD 9.1; *P*=.009) and 6 months (mean 46.04, SD 10.9 vs mean 40.17, SD 9.9; *P*<.001). Although the QoL scores in the control group were lower than those in the experimental group, no significant differences were found between the 2 groups at 2 months (mean 0.75, SD 0.1 vs mean 0.77, SD 0.2; *P*=.11) and 6 months (mean 0.76, SD 0.3 vs mean 0.79, SD 0.2; *P*=.07).

**Table 3:** Comparison of resilience, depressive symptoms, and quality of life in the experimental and control groups (N=103).

Outcomes measures		Experimental (n=52), mean (SD)	Control (n=51), mean (SD)	*t* test (*df*)^a^	*P* value	Experimental (n=52), median (range)	Control (n=51), median (range)	*Z*	*P* value
**Resilience**									
	Baseline	63.48 (15.1)	61.73 (14.7)	–0.598 (101)	.55	63 (32-96)	62 (18-84)	–0.376	.71
	2 months	69.35 (13.4)	61.90 (14.6)	–2.673 (101)	.005	69 (31-96)	62 (12-94)	–2.846	.004
	6 months	67.96 (15.8)	58.27 (19.0)	–3.521 (101)	<.001	66 (41-100)	58 (22-87)	–3.102	.002
**Depressive symptoms**									
	Baseline	45.40 (7.7)	44.16 (7.1)	–0.852 (101)	.40	45 (30-67)	44 (29-70)	–0.740	.46
	2 months	40.40 (9.1)	44.66 (8.0)	2.554 (101)	.009	39 (24-62)	45 (24-71)	–2.665	.008
	6 months	40.17 (9.9)	46.04 (10.9)	3.467 (101)	<.001	40 (25-63)	45 (31-64)	–3.025	.002
**Quality of life**									
	Baseline	0.77 (0.1)	0.76 (0.1)	–0.191 (101)	.85	0.82 (0.41-1.00)	0.78 (0.44-0.96)	–0.801	.42
	2 months	0.77 (0.2)	0.75 (0.1)	–1.589 (101)	.11	0.82 (0.49-0.96)	0.76 (0.45-1.00)	–1.456	.15
	6 months	0.79 (0.2)	0.76 (0.3)	–1.791 (101)	.07	0.81 (0.47-1.00)	0.79 (0.48-1.00)	–1.340	.18

^a^The significance of *t* test is 2-tailed.

### Generalized Estimating Equation Model

There were 16.5% (17/103) of cases with missing data on the variables during follow-up, including resilience, depressive symptoms, and QoL. Table 4 shows the results of the generalized estimating equation model for study outcomes under the intention-to-treat and complete case analyses. Similar results between the 2 analyses reflected the robustness of multiple imputation. Statistically significant main effects were found for the intervention in promoting resilience (*β*=6.082; *P*=.01) and decreasing depressive symptoms (*β*=−2.772; *P*=.04), whereas no significant intervention effect was observed for QoL (*β*=.020; *P*=.38). In addition, significant group-by-time interaction effects were detected for resilience at 2 months (*β*=5.812; *P*=.01) and 6 months (*β*=7.167; *P*=.01) and for depressive symptoms at 2 months (*β*=−5.553; *P*<.001) and 6 months (*β*=−6.504; *P*<.001). No significant interaction effects were observed for QoL at 2 months (*β*=.021; *P*=.42) or 6 months (*β*=.023; *P*=.43).

**Table 4:** A generalized estimating equation model for resilience, depressive symptoms, and quality of life.

Outcome measures			Intention-to-treat (N=103)					Complete case (N=86)		
			*β* (SE; 95% CI)		*P* value		*β* (SE; 95% CI)			*P* value
**Resilience**										
	Main effect^a^	6.082 (2.360; 1.455 to 10.709)		.01		6.055 (2.652; 0.858 to 11.253)			.02	
	Group×time 1^b,c^	5.812 (2.363; 1.177 to 10.448)		.01		6.373 (2.226; 2.011 to 10.736)			.005	
	Group×time 2^b,c^	7.167 (2.921; 1.436 to 12.899)		.01		7.605 (2.739; 2.236 to 12.974)			.004	
**Depressive symptoms**										
	Main effect	−2.772 (1.354; −5.427 to −0.117)		.04		−2.454 (1.581; −5.553 to 0.644)			.12	
	Group×time 1	−5.553 (1.233; −7.971 to −3.135)		<.001		−5.403 (1.177; −7.710 to −3.096)			<.001	
	Group×time 2	−6.504 (1.592; −9.633 to −3.375)		<.001		−7.251 (1.417; −10.028 to −4.474)			<.001	
**Quality of life**										
	Main effect	.020 (0.023; −0.025 to 0.064)		.38		.014 (0.025; −0.035 to 0.063)			.58	
	Group×time 1	.021 (0.026; −0.030 to 0.073)		.42		.021 (0.027; −0.032 to 0.074)			.45	
	Group×time 2	.023 (0.030; −0.035 to 0.082)		.43		.021 (0.027; −0.032 to 0.073)			.44	

^a^Referred to control group.

^b^Referred to control group and baseline.

^c^Time 1 and time 2 refer to 2 and 6 months of follow-up, respectively.

## Discussion

### Principal Findings

This RCT addressed an important health issue related to the consequences of children’s cancer on their parents’ psychological well-being and QoL, an area that has not been well explored. Most importantly, this study is original and helps to clarify the effectiveness of a mobile device–based resilience training program in reducing depressive symptoms and enhancing resilience and QoL in Chinese parents of children with cancer. To our knowledge, this study is the first RCT that combines mHealth and resilience training to improve mental health in the parents of children with cancer.

This study demonstrated that over a 2-month period, this mobile device–based resilience training program was sufficient to enhance resilience in the parents of children with cancer. The findings were in accordance with those of a previous study conducted in students using a computer device compared with educational attention control [38]. Guided by the resilience framework of Kumpfer [24], our program provided training in skills to foster internal resilience factors and enhance the resilience levels of parents of children with cancer. For example, the practice of recording 3 good things every day could encourage the participants to focus on positive things so as to cultivate positive emotions and optimism related to internal emotional characteristics [39]. Cognitive reframing training, which usually involves internal cognitive characteristics, could help the participants view the situation from various perspectives and change their negative thoughts into positive ones, thus allowing them to deal with trouble in cognition [40]. Moreover, to foster internal spiritual characteristics, the participants were encouraged to express their gratitude in various ways to those who supported them. Such gratitude practices have been shown to cultivate participants’ positive beliefs and help them attain personal growth [41]. In addition, the difference in resilience between the experimental and control groups was still significant after 6 months of follow-up, which indicates that the effectiveness of a mobile device–based resilience training program can be sustained over an extended period. Increased proficiency in skills use may play a role in maintaining resilience levels and help parents better navigate adversity [42].

The mobile device–based resilience training program significantly alleviated depressive symptoms in parents of children with cancer. The depressive symptoms score increased continuously across the study for the parents in the control group, whereas the score for the parents in the experimental group showed an opposite trend. These findings were consistent with those of previous resilience training programs for patients with chronic illnesses [16] and contrary to the changes in resilience in this study. The negative relationship between resilience and depressive symptoms has been widely acknowledged [33,43], and our findings further support this relationship in parents of children with cancer. It has been noted that resilience can protect individuals from stress-related disorders such as depressive symptoms via positive cognition and enhanced self-efficacy [44]. Individuals with high levels of resilience are more likely to believe that they have the skills to deal with an adverse situation and to appraise the situation as a challenge to overcome [45]. In this study, problem-solving skills were taught to help parents deal with the issues they encountered in caring for their children with cancer. Combined with character strength training, parents were expected to use their cultivated strengths to solve related problems in daily life. Hence, the learned skills that could increase parents’ confidence in coping with their children’s cancer may have a positive effect on alleviating their depressive symptoms. Furthermore, evidence has shown that relaxation techniques and strong relationships are beneficial for ameliorating depressive symptoms [46,47]. The guided breath awareness practice and parent–child communication training included in the resilience training program may also play a role in reducing depressive symptoms in parents of children with cancer.

Although the QoL scores in the experimental group were higher than those in the control group, no significant effects were detected. The improvements in resilience and alleviation of depressive symptoms did not significantly promote QoL in the parents of children with cancer, possibly because of the relatively short study period. It has been well demonstrated that a change in QoL usually takes a longer time than behavior changes or mental health improvement [48]. Previous studies generally included 3 months of follow-up to explore the effect of a resilience training program on QoL, and the pooled results of the systematic review showed no significant improvement [16]. It is recommended that future studies add further evidence on the long-term effects of a mobile device–based resilience training program on QoL in parents of children with cancer. In addition, QoL is regarded as a multidimensional concept that is influenced by various factors other than resilience and depressive symptoms [49,50]. The skills trained in this study focused mainly on promoting the parents’ psychological well-being, and strategies that target the cultivation of social resources and improvement of physical health are worth considering in future interventions. Evidence has shown that COVID-19 is a stressful event for the general public and can have an impact on their QoL [51]. Given that the study period covered the first wave of COVID-19 in China, the pandemic might have had an impact on the parental QoL and weakened the intervention effects. However, the efficacy in enhancing resilience and reducing depressive symptoms indicated that the learned skills in resilience training could help parents adapt to adversities, including the pandemic.

### Limitations

There were 2 limitations to this study. First, we only examined the effects of the mobile device–based resilience training program on parents of children with cancer for 6 months. Whether the improvement in resilience and the reduction in depressive symptoms could be sustained or whether the changes in QoL could show a significant difference over the long term should be further explored. Second, because of the small sample size, we did not perform a statistical assessment to identify the potential impacts of social demographics and children’s clinical characteristics (ie, time since diagnosis) on parents’ outcomes. Nevertheless, we conducted a cross-sectional study to explore the relationships between resilience and QoL in Chinese parents of children with cancer [52] before this RCT. The results showed that the time since diagnosis did not correlate with parents’ resilience and QoL.

### Implications for Future Practice and Research

Given the increasing incidence of childhood cancer and considerable stress in caring for these children [1], it is critical in clinical practice to provide effective psychological interventions for parents of children with cancer. Our findings indicate that our mobile device–based resilience training program was efficient in enhancing resilience and reducing depressive symptoms. As a potential preventive strategy for stress-related disorders [14], the application of a resilience training program is recommended at an early stage when parents first face the diagnosis of their child’s cancer. The learned skills would then promote parental resilience and help them adapt more quickly to their child’s cancer and deal with the adversity without the harassment of stress-related disorders. Considering the effectiveness and convenience of the mobile device–based resilience training program, health care professionals could use popular social media apps such as WeChat, Facebook, and WhatsApp to implement such programs to benefit more parents of children with cancer via mHealth care. As objective biometric data can complement the subjective evidence of the effects of the mobile device–based resilience training program, relative biometric outcomes should be assessed in future studies. In addition, given that changes in children’s health conditions might affect the parents’ outcomes, it is recommended that future studies also assess the health condition of children with cancer over time. Finally, the parental physical symptoms, such as insomnia, could be good indicators of the effectiveness of resilience training programs, which should be assessed in future studies.

### Conclusions

Our mobile device–based resilience training program was developed under the resilience framework of Kumpfer [24] and included 8 tweets and assignments to train skills that foster internal resilience factors. The parents of children with cancer who participated in this program revealed higher levels of resilience and fewer depressive symptoms than the control subjects. It is highly recommended that health care professionals incorporate this resilience training program when providing psychological care to parents of children with cancer.

## Appendix

Multimedia Appendix 1 English text version of 8 tweets in the mobile device–based resilience training program.

Multimedia Appendix 2 CONSORT-eHEALTH checklist (V 1.6.1).

## Abbreviations

CONSORT: Consolidated Standards of Reporting Trials
mHealth: mobile health
QoL: quality of life
RCT: randomized controlled trial
