# Supplementary material for: Effectiveness of a Mobile Device–Based Resilience Training Program in Reducing Depressive Symptoms and Enhancing Resilience and Quality of Life in Parents of Children With Cancer: Randomized Controlled Trial
Source: J Med Internet Res. 2021 Nov 29;23(11):e27639. doi: 10.2196/27639 (PMC8669578; doi:10.2196/27639)
Supplement: Multimedia Appendix 1 [file jmir_v23i11e27639_app1.docx]

**Supplementary material**

English text version of the eight tweets in the mobile device-based resilience training programme

**Week 1 Introduction**

The purpose of this resilience training programme is to provide emotional and behavioural management and parent-child relationship training for the parents of children with cancer to help promote their well-being.

The main target of this programme is psychological resilience, which refers to the process of adapting well in the face of adversity or stress. After it is squeezed, a sponge automatically returns to its original state, which is the resilience possessed by a sponge; after experiencing adversity, an individual’s psychological condition can also be restored or even improved by cultivating his or her own psychological resilience.

Resilience training guided by positive psychology theory has been widely applied in students and patients with chronic illnesses and has achieved positive results. In life, people often pay too much attention to negative things and emotions but ignore the beautiful things around them. Positive psychology uses scientific principles and methods to guide people to pay attention to positive things to promote a positive emotional experience, cultivate positive personality characteristics and create a positive social environment.

Based on the positive psychology theory and previous investigations, our research team developed a resilience training programme for the parents of children with cancer; this programme includes eight sections: introduction, relaxation, problem-solving skills, character strength training, cognitive strategies, parent-child communication, gratitude practice and summary. Each tweet includes one section, and one tweet will be sent every Saturday. The parents of children with cancer are encouraged to read the tweet sessions and finish the online assignments, each of which should take approximately 15 minutes to finish. Feedback on the assignment from a psychological consultant will be sent back to the parents via WeChat. This programme will last 8 weeks.

***Assignment –*** ***recording three good things***

Please record three good things that happened today. They can be important or trivial. As long as it is something that makes you feel positive, you can record it and analyse the background of the incident and the reason that you are happy.

*An example for reference*

Thing 1: I bought Huanhuan bananas today; she really likes to eat bananas.

Background: Bananas have always been Huanhuan’s favourite fruit. Recently, due to chemotherapy, Huanhuan’s appetite has been poor. I want to give her something she likes to improve her appetite.

Reason for happiness: The bananas are very fresh, and Huanhuan is happy to eat. If her appetite improves, her nutrition can be guaranteed, which is good for her recovery.

**Week 2 Relaxation**

Meditation is the core technique in mindfulness training, which was established in 1979 by Jon Kabat-Zinn. Its purpose is to teach individuals to use their inner strength to actively do things for their own health that others cannot replace.

Mindfulness refers to a conscious and uncritical focus on observing the present moment. This attention can touch the tranquillity of our hearts, help us accept the current situation and keep our heads clear, so that we can deal wisely with the challenges of daily life.

Meditation refers to the enhancement of self-awareness and well-being by achieving deep tranquillity. When meditating, you are completely awake, but the brain does not pay attention to the external world or surrounding things. Meditation maintains inner focus, clarity and relaxation.

Many studies have shown the positive results of mindfulness training, such as enhancing immunity in the general population, increasing the activity of the left front part of the brain that is responsible for positive emotions, and reducing pain in cancer patients and work stress in company employees.

Specific methods to practice meditation (shown in the video)

The first thing you need to do is choose an object of attention for yourself; it can be a sound, a word or your own breathing, body sensation or movement sensation. After selecting the attention object, all you need to do is sit comfortably, close your eyes and perform a simple abdominal breathing relaxation exercise (no more than 1 minute). Then, adjust your breathing to focus on the selected attention object. In the process of training, some other thoughts or feelings may appear in your mind that cause your attention to shift; it does not matter, just return to the original attention. No matter what thoughts appear in your mind, you don’t have to worry. You simply need to return your attention to your breath; don’t be afraid, don’t regret and don’t feel any judgment. After training like this for 10 minutes, rest quietly for 1 to 2 minutes before engaging in other normal work activities.

There are several techniques for practicing meditation. Do not make judgments, be patient, hold a beginner’s heart, trust yourself, do not force the desired purpose, accept the current situation and let go of all the good and evil.

***Assignment – guided breath awareness***

Location: Choose a quiet place.

Posture: Sit or lie down. For the first practice, it is best to choose a seat with a backrest to make the whole body feel comfortable and relaxed.

Duration: Five minutes each time for the first practice, extending to 10 minutes after proficiency.

Content: Please use the audio included in this tweet to practice meditation according to the instruction.

**Week 3 Problem-solving skills**

Problem-solving skills allow people deal with difficulties and actively solve daily problems through a variety of strategies and methods. Many studies of the parents of children with cancer have shown the effectiveness of problem-solving therapy in improving the parents’ well-being.

First, let us look at two different beliefs for solving problems. One is the positive thoughts we should have. This is mainly manifested in the following aspects. (1) Tell yourself that the problem is a challenge, not a threat. (2) Believe that the problem can be solved. (3) Have confidence in your ability to solve the problem. (4) Make efforts to solve the problem. The other is the negative thoughts we should try to avoid, such as being frustrated and choosing to escape before trying to solve the problem.

Seven steps to solve a problem

Step 1: Recognise the problem from five aspects. Who is involved in this problem? What happened? When and where does this happen often? What methods have you tried to solve this problem? Divide the problem into several small pieces and solve them one by one.

Step 2: Set a goal. The goal should be specific, measurable, achievable, reasonable and time limited.

Step 3: Propose solutions. The principles of proposing solutions are as follows. (1) Think of as many solutions to the problem as possible. (2) Write down all the ideas and methods to solve the problem, the more the better. (3) There is no need to evaluate the feasibility or the pros and cons of the methods while listing the methods.

Step 4: Weigh the pros and cons. The principles of weighing pros and cos are as follows. (1) Evaluate each solution based on your own actual situation. (2) Assess the funds and time needed. (3) Consider the short-term and long-term effects.

Step 5: Choose the optimal solution. The principles of choosing the optimal solution are as follows. (1) Do not worry and take it easy step by step. (2) If you are in a dilemma, you can rank the importance of the pros and cons of each solution. The option with the most advantages is the optimal solution. (3) If necessary, you can seek help from others.

Step 6: Establish an action plan. The principles of establishing an action plan are as follows. (1) There are clear steps for implementing the optimal plan. (2) Execution of the plan cannot guarantee the results, but the important thing is that these steps guide you towards the goal. (3) If your execution is not smooth, you may need to reconsider the plan or choose another solution to establish an action plan.

Step 7: Implement and evaluate. If everything is successfully completed, move on to the next problem or goal. If the result is not as good as you desired, evaluate where the effect is not achieved in the process and repeat the previous six steps.

***Assignment – problem solving practice***

Please list a problem you encountered during the past week and apply the seven steps of problem-solving therapy to solve it.

**Week 4 Character strength training**

Character strength refers to the positive characteristics of personality, a series of positive personality traits that are manifested via individual thoughts, feelings and behaviours. Various character strengths can form a higher level of virtue, which is the core element of human happiness and health.

To help people understand their own character strengths through scientific methods, the psychologists Christopher Peterson and Martin Seligman developed a character strength assessment system. The formulation of this system is guided by the canons of the three civilisations of the world’s history and the virtues that are respected in each culture: the ancient Indian civilisation; the Chinese civilisation based on the Tao of Confucius and Mencius and Lao Tzu’s *Tao Te Ching*; and the Mediterranean civilisation based on the Bible and on ancient Greek and Roman philosophy. The character strength assessment system refined 24 character strengths and divided them into six core virtues. The core virtues can be obtained by cultivating the 24 character strengths.

Character strength training is as follows.

Step 1: Determine your own character strengths or advantages through scientific methods.

(A link is provided to the character strength assessment tool.)

After completing all the questions, the system will automatically analyse your strengths and weaknesses. According to the assessment report, everyone can determine their five major character strengths or advantages.

Step 2: Analyse which aspects of your five major advantages are manifested in daily life and work.

(A detailed description of the 24 character strengths is provided.)

Step 3: Think about how to use your five advantages in your future life.

***Assignment*** ***– character strengths training***

Please take a screenshot of your five major advantages after completing the character strength assessment. Then, select one advantage and analyse the ways in which the advantage is manifested. Finally, give an example of how to use this advantage in your future life.

*An example for reference*

The screenshot shows that one of the five major advantages is ‘gratitude’.

Gratitude is mainly manifested in the following ways. Many friends and relatives have visited our child in the hospital, and I am grateful that so many people care about me. During the difficult time when my child was first diagnosed, my mother accompanied me, comforted me and supported me in the hospital every day. I am very grateful to her.

Future use in life: In the ward, the parents usually help each other. When others help us, I will sincerely thank them. I will also help others according to my own abilities.

**Week 5 Cognitive strategy**

Cognition refers to an individual’s view of things. This view can influence the individual’s internal psychological activities and lead to the generation of emotions and behaviours. Therefore, cognition plays a vital role in changing our emotions and behaviours. In psychology, scientists created cognitive therapy based on the importance of cognition. The purpose of this therapy is to help individuals change incorrect cognitions, thus eliminating bad emotions and behaviours. The cognitive restructuring strategy we introduced today is based on cognitive therapy.

Cognitive therapy guides us to understand and think about problems from various aspects and help us divide the event into small parts, including thoughts, emotions, physical sensations and behaviours. Each part may affect other parts. How you look at the event may affect your emotions and physical feelings and may also change your corresponding behaviour. In most cases, based on your views or understanding of the event, you can react in a beneficial way or an unhelpful way. A scenario is provided to show the effects of various thoughts on emotions, physical sensations and behaviours.

Scenario: You need to take your child to the hospital regularly for chemotherapy.

|  | Unhelpful | Beneficial |
| --- | --- | --- |
| Thoughts | Every time my child undergoes chemotherapy, it is painful, why should my child suffer so much pain? | The child used to go to the hospital if she caught a cold. Treat it as a cold – a cold once a month. |
| Emotions | Depressed, sad, self-blaming | Positive emotions, optimism |
| Physical feelings | Insomnia, tiredness | No special feelings, comfortable |
| Behaviours | Blame others, dare not face the child’s treatment. | Take care of the child carefully. |

Tips for emotion management

1. Attention shifting refers to turning one’s attention away from situations that can easily cause bad emotions. Certain activities can be done to divert one’s attention, such as hiking and running.
2. Expression suppression refers to the suppression of emotional expression that is about to occur. You can use deep breathing to calm yourself.
3. The social conversation strategy is to encourage people to communicate with others, such as families, friends, colleagues and the parents of other children in the ward.

***Assignment – cognitive restructuring practice***

Please think of a recent situation or event that caused bad emotions. Describe your thoughts at that time. What kind of physical feelings did it produce? What kind of behaviours did it cause? Please give a new and beneficial thought of the situation or event, and then record the emotions, physical feelings and behaviours that came along with it.

**Week 6 Parent-child communication**

Parent-child communication refers to communication between a parent and a child. The purpose of parent-child communication is not only to transmit information, but more importantly, to establish a harmonious relationship.

Methods to achieve effective parent-child communication are as follows.

1. Emotion management. Learning to manage emotions is the first step to effective communication. Parents should learn to control their own emotions and try not to lose their temper in front of the child. The skills to manage emotions have been mentioned in the previous sections.
2. Appropriate expression. When communicating with the child, parents should express their feelings constructively and give some appropriate explanations. Do not use invalid means such as requests, orders and blame.
3. Empathy. Parents should think about and look at things from the child’s perspective and should not impose their own preferences on the child. When the parents understand the child’s emotions, they can respond appropriately.
4. Positive listening. If parents wish to understand their child’s inner thoughts, they need to allow the child to express them and should listen to the child’s voices. When talking with the child, avoid interrupting the child’s speech so that the child feels respected. Allow children to have their own ideas and opinions.

Five steps to manage the child’s emotions are as follows.

Step 1: Learn to observe the child’s emotions. Sadness, fear, anger, happiness, curiosity and love are common emotions in children with cancer.

Step 2: Provide the child with an open, tolerant and relaxing space where the child can express his or her feelings freely.

Step 3: Show understanding of the child’s emotions, accept the child’s feelings, affirm his or her value and finally provide advice or help.

Step 4: Help the child recognise his or her own emotions by using pictures or telling stories or appropriate communication.

Step 5: Help the child establish ways to release their emotions, such as outdoor activities and relaxation practice.

An example of effective parent-child communication (shown in the video)

‘Whenever you’re feeling moods like this (anger), you shouldn’t just stay there all the time. You can accept and acknowledge it, and then let it go, and it’ll get better. And if it doesn’t, you don’t make it, just let it be. You’re still loved, and you’re surrounded by white light. It’s ok to feel this way. You can be mad, just don’t hang on to it too long, because that’s when it gets to be a problem. Let it go and work through it, and sometimes you can just stop, or yell, or punch a pillow’.

***Assignment – parent-child activity***

Please accompany your child with an appropriate activity, such as making handcraft with little children and engaging in moderate outdoor exercise with teenagers.

**Week 7 Gratitude practice**

Gratitude can promote physical and mental health. Psychologically, gratitude allows us to experience more positive emotions, including pleasure, love and happiness, and to feel more satisfied with life. Physically, people who know how to be grateful feel less physical discomfort and are relatively more willing to spend time exercising. Gratitude can also promote the establishment of interpersonal relationships and stimulate behaviours that are beneficial to others.

Psychologists have developed gratitude training and conducted a series of studies that showed that college freshmen showed better adaptation to college life after a 3-week gratitude training programme. An Internet-based survey showed that 3-month gratitude diary training can improve individuals’ happiness and reduce depression.

Ways to practice gratitude are as follows.

1. Gratitude diary: record the things for which you are grateful in your daily life in the form of a diary.
2. Gratitude letter: write a letter about the things you want to show thanks for and give it to the person you wish to thank.
3. Gratitude visit: visit someone to whom you are grateful and express your gratitude in person.
4. Gratitude card: write a thank-you note on the card and give it to the person you want to thank.
5. Gratitude call: make a call to someone to whom you are grateful and express your gratitude on the phone.

The principle of goal setting: specific, measurable, achievable, reasonable and time limited. When setting goals, one must first recognise one’s current situation and make a list of goals that can be achieved this week (or this month). Then classify the goals and sort them in order of priority, and write a planned completion time after each goal. Finally, take specific actions.

An example of goal setting from parents of children with cancer.

‘I’ll buy whatever she (the child) wants to eat. If she says she wants to eat any fruit, I’ll buy it. I have such a small goal every day, I’m happy. I’m grateful to live this life every day, after having experienced the period of torment in the hospital. Everyone is equal in the face of illness, so now we need to live every day. Let the child be happy, let everyone in the family be happy, I’ll do whatever I want, I set a small goal and then achieve it on the day. Because I don’t know what it will be like tomorrow or in the future. It can only be like this, and I think it’s pretty good’.

***Assignment – gratitude practice***

Please choose one of the ways to practice gratitude, including a gratitude diary, letter, visit, card or call. The object of gratitude can be a family member, a friend, a colleague, or a medical staff member. The things for which you are grateful do not need to be very important, as long as you feel that they have meaning for you.

**Week 8 Summary**

The resilience training programme consists of eight tweet sessions: introduction, relaxation, problem-solving skills, character strength training, cognitive strategies, parent-child communication, gratitude practice, and summary. The eight targeted assignments were recording three good things, guided breath awareness, problem-solving practice, character strength training, cognitive restructuring practice, parent-child activity, gratitude practice, and plan-making.

Below, a simple review of the previous tweet sessions is presented.

*Session 1*: Pay attention to the beautiful things around you and cultivate positive emotions and a positive personality. The assignment is to record three good things and to think about the background of what happened and why you are happy.

Frequency: Once a day, at least five times a week.

*Session 2*: Meditation is about making friends with your own thoughts; learning to understand your emotions, thoughts and behaviours; and finding peace under waves of anxiety. The focus of meditation practice is to find your own comfortable posture, discard all distracting thoughts, and focus your attention on an object of your choice, which can be your breathing or a word or a sound.

Frequency: Once a day, 5 to 10 minutes each time.

*Session 3*: The seven steps to solve problems are to recognise the problem, set a goal, propose solutions, weigh the pros and cons, choose the optimal solution, establish an action plan and implement it. Have a positive belief that the problem can be solved. When proposing solutions, we can seek support from others and propose as many solutions as possible. Frequency: Once a week, solve one problem each time.

*Session 4*: Discover advantages with the character strength assessment system, and analyse the specific performance of character strengths in daily life and think about how to apply them in future life.

Frequency: Once a week, analyse and apply one of the five advantages.

*Session 5*: In the cognitive strategy, we exemplified that in the same situation, different thoughts can have different effects on emotions, physical feelings and behaviours. Try to develop positive thoughts about an event and experience the feelings that come along with those thoughts.

Frequency: Once a week, analyse one event each time.

*Session 6*: We suggest that parents adjust their own emotions, actively listen in the process of communication with the child, express their feelings constructively and learn to think from the child’s perspective. Accompany the child in completing a parent-child activity and apply communication skills in the activity.

Frequency: Twice a week, at least ten minutes each time.

*Session 7*: Gratitude can promote well-being and make the world better. This section introduced various forms of gratitude exercises, including gratitude diary, letter, visit, cards and calls.

Frequency: Once a week.

***Assignment – plan-making***

Please choose one to four assignments that you like and make a 1-month exercise plan.
